# A New Association Approach for Multi-Sensor Air Traffic Surveillance Data Based on Deep Neural Networks

**DOI:** 10.3390/s25030931

**Published:** 2025-02-04

**Authors:** Joaquin Vico Navarro, Juan Vicente Balbastre Tejedor, Juan Antonio Vila Carbó

**Affiliations:** 1 Instituto Universitario de Automática e Informática Industrial, Universitat Politècnica de València, Camí de Vera s/n, 46022 Valencia, Spain; 2 Instituto Universitario de Tecnologías de la Información y Comunicaciones, Universitat Politècnica de València, Camí de Vera s/n, 46022 Valencia, Spain

**Keywords:** performance monitoring, performance-based surveillance, machine learning, neural networks, opportunity traffic, aeronautical surveillance

## Abstract

Air Traffic Services play a crucial role in the safety, security, and efficiency of air transportation. The International Civil Aviation Organization (ICAO) performance-based surveillance concept requires monitoring the actual performance of the surveillance systems underpinning these services. This assessment is usually based on the analysis of data gathered during the normal operation of the surveillance systems, also known as opportunity traffic. Processing opportunity traffic requires data association to identify and assign the sensor detections to a flight. Current techniques for association require expert knowledge of the flight dynamics of the target aircraft and have issues with high-manoeuvrability targets like military aircraft and Unmanned Aircraft (UA). This paper addresses the data association problem through the use of the Multi-Sensor Intelligent Data Association (M-SIOTA) algorithm based on Deep Neural Networks (DNNs). This is an innovative perspective on the data association of multi-sensor surveillance through the lens of machine learning. This approach enables data processing without assuming any dynamics model, so it is applicable to any aircraft class or airspace structure. The proposed algorithm is trained and validated using several surveillance datasets corresponding to various phases of flight and surveillance sensor mixes. Results show improvements in association performance in the different scenarios.

## 1. Introduction

Air Traffic Surveillance (ATS) relies on a heterogeneous network of sensors of different technologies: Automatic Dependent Surveillance-Broadcast (ADS-B), Primary Surveillance RADAR (PSR), Secondary Surveillance RADAR (SSR), Surface Movement RADAR (SMR) or Ground Radar, and Wide Area Multilateration (WAM) [1]. The Performance Based Communication and Surveillance (PBCS) concept requires all Air Navigation Service Providers (ANSPs) to evaluate the performance of their infrastructure of surveillance sensors. The PBCS manual [2] provides objective operational criteria to perform this task based on a set of performance-based specifications, for surveillance and communications known as Required Surveillance Performance (RSP) and Required Communications Performance (RCP). The PBCS concept also includes post-implementation Surveillance Performance Monitoring (SPM) programmes for continuous assessment of the system. The evaluation consists of an off-line process where sensor data are compared to *reference trajectories.* A *reference trajectory* is the best estimate of the aircraft track that results from fusing the information coming from all sensors.

An aircraft trajectory is described as a *track*, usually implemented as a list of *target reports*. Each target report contains the basic aircraft data: identification, position, altitude. In some cases, kinematic information is also available. However, not all sensors provide this information: some sensors do not provide altitude or aircraft identification, and some other sensors are only available in some parts of a track. This is the case of RADARs, which usually are only available in some Terminal Maneuvering Areas (TMAs). Additionally, each sensor technology has its own performance features, like accuracy and error models. So, sensor fusion needs to address all these issues.

This paper aims to improve some of the stages in the calculation of the optimal reference trajectory. This calculus is embodied in several tools to used for Performance Monitoring (PM), with openATS COMPASS and EURCONTROL Surveillance Analysis Support System for ATC Centres (SASS-C) [3] being the most used in European airspace. These tools use opportunity traffic gathered by ATS and usually rely on Bayesian filtering processes, namely Kalman Filtering and Nearest Neighbour algorithms [4]. This paper presents the insights gained by the authors during the re-engineering process of the SASS-C VERIF module.

The process for generating reference trajectories usually consists of two stages: an association and a fusion process. In SASS-C, these stages are referred to as *Target Association*, and *Trajectory Reconstruction*. This paper only deals with the first phase: the Target Association process of SASS-C.

Most tools currently used for Performance Monitoring (PM) are model-based: they take advantage of sensor and aircraft features and their equations of motion. They perform well when the airspace is nearly homogeneous, such as in the cruise phase of commercial aviation. But their performance decreases when different types of targets are considered, such as Unmanned Aerial Vehicles (UAVs), general aviation and military aviation [5].

Target Association in sensors that provide a unique identifier of the originating target, like ADS-B is straightforward. For sensors not providing this identification, the association is model-based: it is performed on spatio-temporal checks that rely on target dynamics (equations of motion). If the report does not meet the checks of any target, the report can be left as *unassociated*. This may happen with outliers or when the sensor performance is lower than required. In  [6], the authors showcase this issue with the association performance of SASS-C with different RADAR sensors, displaying an unassociated measurement rate of close to 50% for PSR sensors, while SSR sensors remained below 2%. This means that almost half of the PSR measurements will not be assessed during the evaluation. It may also happen that a report is associated to a wrong target, yielding a *miss* or a false association, also known as a mismatch. This can occur when reports of several targets are close together, as in the case of trajectory crossing or flying in formation. Model-based association also performs poorly with rapid changes in Mode of Flight (MoF), such as take-off and landings for commercial aviation and turns and accelerations from high-manoeuvrability aircraft such as military aircraft. These drawbacks can significantly degrade the quality of reference trajectories and, in consequence, the accuracy of the PM.

This paper introduces a novel approach for the Target Association process called the Multi-Sensor Intelligent Data Association (M-SIOTA) algorithm. It is an association algorithm based on a Deep Neural Network (DNN) trained with opportunity traffic to group detections into tracks. The main advantage of M-SIOTA is its ability to perform this task without any prior knowledge about the target, the current set of sensors used, or the airspace environment, relying only on previously recorded opportunity traffic. This way, it does not require expertly tuned models for each aircraft type in the airspace, thus reducing the effort required to adapt to new target dynamics. It implements a novel architecture based on the newer surveillance technologies available (such as ADS-B) and leverages Machine Learning (ML) techniques to provide several advantages over conventional model-based algorithms. The main advantages of the algorithm include the ability to deal with different types of traffic (commercial, general, military, UAVs, etc.) without the development of specific kinematic models; an improved prediction performance with rapid changes in trajectory dynamics [5]; a reduction in the complexity of the tools used for opportunity traffic analysis; and incremental improvements in performance as more data are available, adapting to changes in the airspace.

This requires extensive training on opportunity traffic-reflecting situations where model-based association usually fails. However, although training can be a costly process, once the algorithm is trained it is quite efficient during execution.

The contributions of this paper are twofold: on the one hand, a new association process is proposed that improves on model-based algorithms used by traditional tools and, on the other hand, it sets the base for a redesign of current tools to introduce Artificial Inteligence (AI)-based methods. This approach is trained and validated using real-world data to evaluate its effectiveness in different scenarios containing common use cases for PM.

This paper is structured as follows. First, Section 2 reviews related work. In Section 3, the M-SIOTA algorithm is presented along with the datasets used and the training process. Section 4 provides the results of the application of the proposed model to several real-world datasets. Lastly, conclusions and closing remarks are contained in Section 5.

## 2. Related Work

Opportunity traffic association is a multi-sensor multi-target problem of data association and fusion. In recent decades, several methods and algorithms have been presented to deal with this topic, from the Kalman filter [7] to single hypothesis methods like the Global Nearest Neighbour (GNN) [8], and probabilistic associators capable of simultaneously tracking several targets such as the Joint Probability Data Association (JPDA) [9] or Multi Hypothesis Tracking (MHT) [10]. Probabilistic association methods deal with environments with high clutter and false detections effectively at the cost of computational resources. However, most Air Traffic Management (ATM) surveillance sensors are cooperative and are not affected by clutter. Furthermore, non-cooperative primary radars have very effective clutter suppression systems; thus, even in this case, the effect of clutter can be neglected in the association process.

These algorithms are based on Bayesian theory and require mathematical models to estimate the state of the target and predict future states from the received measurements. The effectiveness of the algorithms is highly dependent on the models and the performance characteristics of the targets, so they require expert tuning. The algorithm presented in this paper tries to avoid these target dependencies and tuning using ML.

Moreover, model-based techniques have proven good results in in-flight and in-ground applications on their own [6,11]. However, there is a lack of association algorithms that can bridge the gap between the flight and ground stages. The proposed algorithm also shows the feasibility of an association algorithm based on DNN that correctly generates tracks that include all sections of the flight.

Previous work on information fusion of multiple sensors uses two main techniques: *track-to-track association and fusion* [12] and *measurement-to-track association and fusion*  [13]. Track-to-track fusion receives as input the local tracks from each sensor, which has its own tracker system by default. On the other hand, *measurement-to-track association* requires raw measurement data from each sensor for the association. Some of the proposed techniques are intended to perform as “near-real-time”, i.e., immediately, as soon as the system is able to perform the high computational cost that they require. RSP assessment does not require real-time performance.

In track-to-track association and fusion [12], the fusion is performed in two steps: the first step is to determine if the tracks are from the same target, while the second step is to combine the associated tracks to form a single estimate. In [14], track-to-track fusion is performed based on Bayesian Minimum Mean Squared Error (MMSE).

Although track-to-track fusion is a computationally efficient approach, estimation errors accumulate over time and, as a result, the overall estimation errors may become large [15]. In addition, fusion performance may degrade due to misassociation across tracks from different sensors [16].

In measurement-level fusion  [13], measurements from different sources are forwarded to the centralized fusion centre and processed jointly by a measurement-to-track association algorithm. In [17], a large-scale air traffic surveillance problem using a centralized architecture is considered. Measurement-level fusion usually offers improved tracking performance over distributed tracking but with the need for more computational resources as well as sufficient bandwidth between the sensors and the fusion centre. Algorithms for maritime surveillance fuse the information of an Automatic Identification System (AIS) and radar measurements. Most of them assume that the identification of the Automatic Identification System (AIS) used in this field is perfect (i.e., no ambiguity). In [15], the authors propose a probabilistic approach based on JPDA to face ambiguity.

Some of the drawbacks of model-based techniques can be overcome using ML techniques in the ATM field. Neural networks have been demonstrated to be universal function approximators [18], avoiding the need for a system model if enough data are available. Recent advancements in data availabilty and training power have pushed the production of data-driven works in the ATM space. In [19], the authors review the current published work on the application of AI to this field and there is a clear focus by the research community on the usage of open data to train machine learning models. These models are used for trajectory prediction [20,21], time of arrival estimation [22,23], delays [24], and aircraft performance  [25]. For position estimation and tracking, the capabilities of ML have been demonstrated in simulated and real single-sensor environments [26,27].

As for multi-sensor environments, there is great interest in the possibilities and applications of ML and AI to data fusion problems [28], with airspace data as one of the prominent application fields. This is, however, difficult because there is a reduced amount of surveillance-level data available for research compared with open ADS-B databases. This adds to the natural challenges of multi-sensor data fusion, mainly differences in error profiles and the amount of information the sensors provide.

In [27], the authors propose a machine learning algorithm for tracking that employs a Deep Long-Short Term Memory (DLSTM) network for estimation. This paper explores a multi-target, single-sensor simulated tracking environment and proves the efficacy of machine learning techniques on this task. However, there are two issues when applying the framework to a multi-sensor association problem: the authors assume a constant measurement rate and use a single sensor with a single noise and bias profile. As stated before, these assumptions are not applicable in a multi-sensor association environment as the sensors in a surveillance network are not synchronised, each with their own sampling rate, and have different error profiles.

In conclusion, higher quality reference trajectories allow the optimization of the surveillance sensor network under PBCS and, while previous tracking and association algorithms remain relevant, there is a need in the aviation community for algorithms that can perform under the different stages of a flight, with multiple heterogeneous sensors, and that do not rely on expertly tuned models.

## 3. Using DNN for Surveillance Data Association

In this section, we present the Multi-Sensor Intelligent Data Association (M-SIOTA) algorithm. First, in Section 3.1, we provide a high-level description of the association process for opportunity traffic. Then, Section 3.2 formulates data association as an optimization problem and proposes a cost function based on DNN. In Section 3.3, we present the M-SIOTA algorithm and network structure of the DNN. Lastly, Section 3.4 and Section 3.5 describe the datasets used and the training process, respectively.

### 3.1. Opportunity Traffic Association

Association systems mainly evolve from online tracking algorithms, as they solve a similar problem: they aim to build and maintain tracks by assigning each sensor detection to a trajectory and estimating the target’s current position. This process is performed separately for each sensor and then merged into multi-sensor tracks for multi-sensor environments such as surveillance.

There is, however, an essential distinction between tracking and association. Tracking algorithms build the tracks sequentially, processing measurements chronologically either one by one or in blocks [29]. For Performance Monitoring use cases, all the measurements from all sensors are available at the time of analysis. This data availability can be leveraged to optimise the association process. A hierarchical approach to sensor processing in a heterogeneous environment allows the tracks to be built from the most reliable information and then supplemented with the rest of the sensors, improving track completeness and reducing error rates [30].

The reference trajectory is later created based on the associated reports with smoothing algorithms [31], often after correcting for sensor bias and noise. In [32], the authors detail the reference trajectory generation process, also known in the literature as reconstruction. The precision and quality of the reference trajectories are highly dependent on the associations performed in the previous stage.

The following paragraphs explain the advantages of using the proposed association process over a classical one used by tools like SASS-C [4].

**Figure 1:**
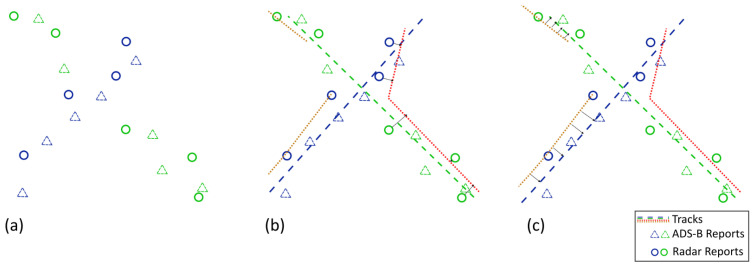
Classical association processes: (**a**) initial reports, (**b**) sensor-by-sensor association, (**c**) track merging.

Figure 1 shows an example of a classical association process with two targets crossing paths. Figure 1a contains the target’s detections before association. The detection’s shape denotes the sensor type and the color denotes the target that originated it. The reports come from different sensors, in this case, PSR and ADS-B. In Figure 1b, single sensor tracks (dashed lines) are created by simultaneously processing the reports independently for each sensor, creating five tracks. Lastly, in Figure 1c the tracks are merged to form multi-sensor tracks. The merging step checks for target identity as well as space–time compatibility [6] and tries to join track pairs that belong to the same target. In this case, the red track will not be merged as it is not compatible with any other track and will compromise the association results, either by creating a *false target* if reports continue to be associated to it or by being dropped due to the short length of the track and resulting in unassociated reports or *misses*.

**Figure 2:**
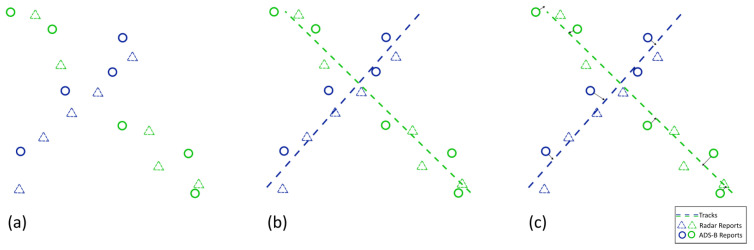
Proposed association process: (**a**) initial reports, (**b**) association of identified reports, (**c**) association of non identified reports.

Figure 2 shows the proposed association process. Instead of relying on the merging of sensor local tracks, the association is performed in a top-down fashion, creating tracks with the reports with the best information (Figure 2b) and enriching them with reports from the other sensors one at a time (Figure 2c). This reduces the number of wrong associations since more information about the target and its trajectory is available when the decision to assign a report to a track is made.

Different sensor technologies provide different information, such as target identity and altitude, and have different accuracies on the position reports [33,34,35]. A hierarchy of sensors is proposed based on two criteria: first, the availability of unique aircraft identification, and second, the positional accuracy.

Mode S capable sensors are considered the best for initial associations. They provide the unique ICAO transponder identification number, or target address, that identifies the target. Upon reception by the respective surveillance sensor, the message is validated to ensure the code was not corrupted during transmission through parity checks [36]. Next in the hierarchy are Mode A capable sensors. They provide aircraft identification (Mode A code); this code is assigned by the ATM controller and set by the pilot in the transponder. Mode A codes are not unique for each aircraft and may change throughout a flight.

Then, the sensors are ordered by positional accuracy, with ADS-B being the most accurate sensor, followed by RADAR and WAM [33,35].

The resulting hierarchy is as follows:

With the proposed approach, the sensors are processed in the order shown in Table 1. Starting with ADS-B, tracks are generated by checking identity and spatiotemporal constraints. Then, the reports are checked sensor by sensor to find a compatible track, associating them to the best candidate. A new track is generated with the report if no compatible track is found.

This approach may generate sparse tracks as the coverage of the different sensors is likely to vary across the flight. This requires *track fusion* after the initial association. Track fusion consists of merging two or more tracks with overlapping segments. This problem is not endemic to this approach, as sequential approaches also have a level of track spuriousness that can be remedied with further processing [37].

**Figure 3:**
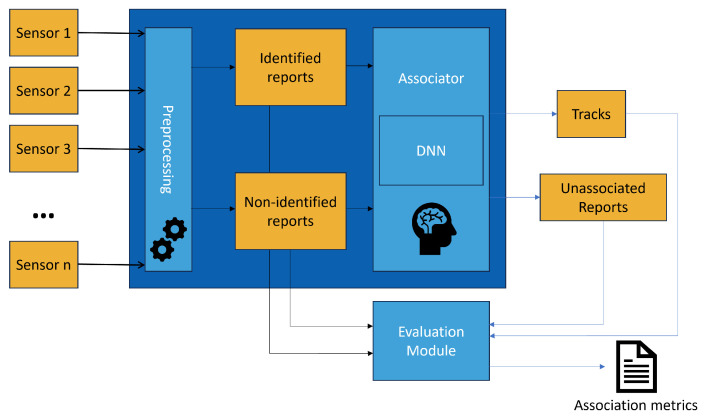
Framework of the proposed association system.

The overall block diagram of the association process set out in this paper is shown in Figure 3.

The main steps of the process are Preprocessing, Association, and Evaluation. In the Preprocessing stage, data are conditioned, and the positions reported by the sensors are projected on a common Cartesian reference plane, as each sensor uses a different coordinate system. Then, the reports are split depending on whether a target address identifies them or not and fed into the Associator that chains the reports into tracks. Lastly, the Evaluation process computes the performance metrics of the association system.

### 3.2. Problem Formulation

In a multi-target tracking scenario, the measurements from the sensors at time step *k* can be represented by the set $Z_{k}=\left\{z_{k1},...,z_{k_{M_{k}}}\right\}$, with $M_{k}$ being the number of measurements for this period. We represent tracks as the set of measurements assigned to a given label $T_{k}^{l_{i}}:\left\{z_{t,\theta_{t}},z_{t+1,\theta_{t+1}},...,z_{k,\theta_{k}}\right\},l_{i}\in N_{k}$, with $N_{k}$ being the number of tracks at time *k*, *t* the first time the track was detected, $\theta_{k}:L_{0:k}\to \left\{0,1,...,N_{k}\right\}$ the association map that describes which track generated each measurement, and $L_{0:k}$ the label space for the tracks. The assignment $\theta_{k}\left(i\right)$ can be equal to 0, meaning that the track *i* does not have a measurement assigned at time *k*.

The association maps for all labels and targets can be expressed as an assignment matrix $A\in R^{N_{k}\times M_{k}}$ with the item $a_{i,j}$ as follows:

(1) $$a_{i,j}=\left\{\begin{array}{cc} 1 & \mathrm{if}\;\mathrm{measurement}\;j\mathrm{is}\;\mathrm{assigned}\;\mathrm{to}\;\mathrm{the}\;\mathrm{track}\;l_{i}\\ 0 & \mathrm{otherwise} \end{array}\right.$$

A value of 0 on a column for a given measurement (*j*) means that no compatible track has been found, and a value of 0 on the row of a given track (*i*) means that no detection is assigned to that track at time *k*. At any time step, a track may only be assigned one measurement, and each measurement may only be assigned to one track, resulting in the conditions in Equations (2) and (3).

(2) $$\sum_{i=1}^{M_{k}}a_{ij}⩽1,a_{ij}=0\;\mathrm{or}\;1$$

(3) $$\sum_{j=1}^{N_{k}}a_{ij}=1,a_{ij}=0\;\mathrm{or}\;1$$

Association cost is commonly defined as the pairwise distance between the predicted state and measurement. In our case, instead of performing state estimation, we replace the cost function with a confidence estimation from the DNN to determine whether the measurement originates from the target, referred to as the CompatibilityScore. Formally, it is defined as $Comp\left(T_{k}^{l_{i}},z_{j}\right)\in \left[0,1\right]$ for the track *i* and measurement *j*. The CompatibilityScore is obtained by training the neural network to minimize the classification error between track and measurement and is explained in detail in Section 3.3.

The items in the association cost matrix $C\in R^{M_{k}\times N_{k}}$ are defined as follows:

(4) $$c_{i,j}=\left\{\begin{array}{cc} -\mathrm{Comp}(x_{k,l_{i}},z_{j}) & \mathrm{if}\;\mathrm{Comp}(x_{k,l_{i}},z_{j})\geq \mathrm{thr}\\ 0 & \mathrm{otherwise} \end{array}\right.$$

The sign of the compatibility score is inversed so the best assignment has the lowest cost and the thr is a threshold to avoid associations measurements with low confidence scores.

The objective of the association algorithm is to calculate the assignment matrix A that minimizes the association cost, which is described as follows:

(5) $$\min_{A}\sum_{i=1}^{M}\sum_{j=1}^{N}c_{i,j}a_{i,j}$$

This is a complex combinatorial optimization problem that can be solved using optimization algorithms such as the Hungarian algorithm. However, this general formulation assumes that there are several measurements in the period under association *k*. In this work, a simplification is made to analyse the measurements one by one, as the multiple sensors may produce more than one measurement from a target if $M_{k}>1$, breaking the condition in Equation (3). Then, the association and cost matrix can be represented as vectors and the assignment of the measurement $z_{k}$ is

(6) $$\left\{\begin{array}{cc} \underset{l_{i}\in L}{\arg\max}\;\mathrm{Comp}\left(T_{k}^{l_{i}},z_{k}\right) & \;\mathrm{if}\;\exists (l_{i}inL|\mathrm{Comp}\left(T_{k}^{l_{i}},z_{k}\right)>thr)\\ 0 & \mathrm{otherwise} \end{array}\right.$$

### 3.3. Multi-Sensor Intelligent Opportunity Traffic Association Algorithm

The core of the proposed algorithm is the ML model that is able to estimate the compatibility between a report from a target and a track, providing a confidence score to the association that can be used to select the best track for the association.

The model developed is based on a DNN for estimation. DNNs and specifically Multi-Layer Perceptrons are powerful tools to infer the underlying characteristics of a system through training on examples of the expected results. These types of networks are widely used for modeless estimation and data fusion [38,39] as well as classification [40].

In our case, the DNN is trained to predict the CompatibilityScore between a track and a report, that is, if they belong to the same originating target. This removes the need for expertly tuned target dynamic models and sensor noise profiles for data association. To predict the compatibility, the model uses the difference between the last *n* measurements in a track and the current report, as defined in Equation (8), and outputs a value in the range [0,1] that represents the model’s confidence in the compatibility between the report and the track. To avoid confusion with other variables, the measurement $z_{k}$ is renamed as *R*.

(7) $$\mathrm{Comp}\left(T_{k}^{l},z_{k}\right)=NN(\Delta \left(T_{k-n+1}^{l},R\right)\;∥\;\Delta \left(T_{k-n+2}^{l},R\right)\;∥\;\cdots \;∥\;\Delta \left(T_{k}^{l},R\right))$$

(8) $$\Delta \left(T_{i},R\right)=\left[\begin{array}{c} x_{T_{i}}-x_{R}\\ y_{T_{i}}-y_{R}\\ z_{T_{i}}-z_{R}\\ t_{T_{i}}-t_{R} \end{array}\right]$$

with $T_{i}$ being the *i*-th measurement in the track $T_{k}^{l}$ and *R* the measurement under consideration; x, y, and y the position of the reports each axis; and *t* the time of applicability of the report.

**Figure 4:**
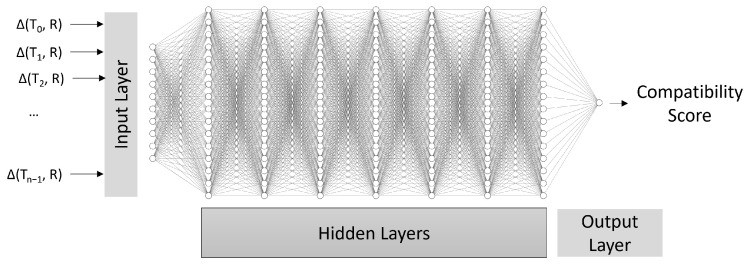
DNN Model Structure.

Figure 4 shows the relationship between the inputs and the outputs of the model.

The model implements a structure based on a sequential set of 10 layer blocks, each composed of a densely connected neural network layer (dense) with 512 neurons each and a dropout layer. The dropout layers can randomly set one of the inputs of a node to 0, with a likelihood of 20% in this case, and are active only during training to prevent overfitting. The dense layers use the ReLU activation function [41].

After those blocks, a last output layer is added, with a single neuron with a sigmoid activation function [42]. This layer outputs a single value in the range [0, 1] used as a metric to signify the model’s confidence in the compatibility of the given track and report. Table 2 summarizes this structure.

The training hyperparameters were tuned using a version of the Hyperband algorithm provided by the Keras libraries version 1.4.6  [43,44]. The resulting parameters after tuning are shown in Table 3.

In order to generate the tracks, the algorithm starts by creating the base tracks from identified data in the dataset. Once the identified reports are associated, the rest of the reports are processed one by one, looking for all the tracks that could be compatible with them and obtaining a CompatibilityScore from each pair of track and report. With that pairwise score, the report is associated with the best match that is above the minimum threshold (0.5). Algorithm 1 describes this process.
**Algorithm 1** Unidentified Report Association
 n=10                     ▹ look-back ammount
 thr=0.5                   ▹ association threshold
 **for** Report **in** non-identifiedReports **do**
     Find all possible Tracks for Report
     **for** Track **in** Tracks **do**
         $prev\leftarrow \{i\in N|0\leq i<\mathrm{length}\left(Track\right)\wedge Track{\left[i\right]}_{t}<R_{t}\}$▹ selecting indices of the previous *n* reports in the track before Report *R*
         $X\leftarrow \Delta_{Track[j],R}|j\in \left[prev,prev-n\right]\}$
         $CompatibilityScore_{Track}\leftarrow DNN_{prediction}\left(X\right)$
     **end for**
     **if** ∃CompatibilityScore≥thr **then**
          Associate Report to Track with highest CompatibilityScore
     **end if**
 **end for**


### 3.4. Dataset Selection

Throughout this paper, several datasets have been used to train and evaluate the model for different applications. Each of the datasets has specific characteristics that present the various challenges found in real ATM environments.

**Availability of ground data**.  Some datasets used have a mix of grounded and airborne aircraft surveillance data. Classical association algorithms have issues when combining ground and air reports, as the movement speeds, turning ratios, and aircraft density are extremely different. For example, the absolute distance between the estimation of a track and the report is commonly used as a criterion for association [4]; however, the acceptable distance threshold for association during flight is greater than the distance between aircraft rolling in an airport.
**Different sensor error profiles**.  Different sensor technologies have different noise and bias profiles. When chaining detections from different sensors, their noise profiles have to be taken into account, which may affect the results of the association.
**Geographical areas**.  Different geographical areas have different flight profiles and obstacles, such as mountains or buildings, that affect the shape of the input trajectories and the performance of the surveillance system. Several different areas were analysed to ensure that the algorithm is robust to these effects.

The datasets used are a monosensor ADS-B dataset from OpenSky, a Surveillance Network dataset with several sensors across a wide area, and a TMA surveillance system with a focus on the airport area. Table 4 contains the main characteristics of the datasets and more details about the different datasets used are provided in the following sections.

#### 3.4.1. Dataset 1: OpenSky

OpenSky is an aggregator of crowd-sourced air traffic control data received through a volunteer network of ADS-B antennas spread across the globe, with high coverage over Europe and North America [45]. They provide datasets for use in scientific publications. The dataset used in this paper is one of the weekly 24 h data, namely the 2022-06-27 dataset (https://opensky-network.org/datasets/states/2022-06-27/ (accessed on 19 October 2023)).

This dataset contains data aggregation from ADS-B receivers worldwide, with a temporal resolution of 1 s. It also includes the coasted estimations of the target position for time steps for which the target did not emit a position report. For the purposes of this paper, coasted detections are filtered out before processing the rest of the dataset.

The main issue of this dataset is that it only has one source, ADS-B surveillance. This limits the model’s potential as one of the aims is to chain detection from different sources with different technologies. Nonetheless, this dataset was used because of the quantity of reports and the possibility of performing evaluations globally.

As the dataset contains reports from flights all over the globe, a selection of an area above Europe was made. This area is delimited by a 20-by-20-degree selection in latitude and longitude over central Europe.

All the reports in this dataset are identified by the ICAO 24-bit identifier. In order to evaluate the performance of the M-SIOTA algorithm, 40% of the identities will be hidden for evaluation as described in Section 4.

#### 3.4.2. Dataset 2: European Surveillance Network

This dataset is a demonstrator with detections across Europe for eight continuous hours from several sensors, namely ADS-B, RADAR, and multilateration surveillance sensors. This dataset contains a reasonably diverse set of flights and several airports. This dataset includes flights from commercial and general aviation.

**Figure 5:**
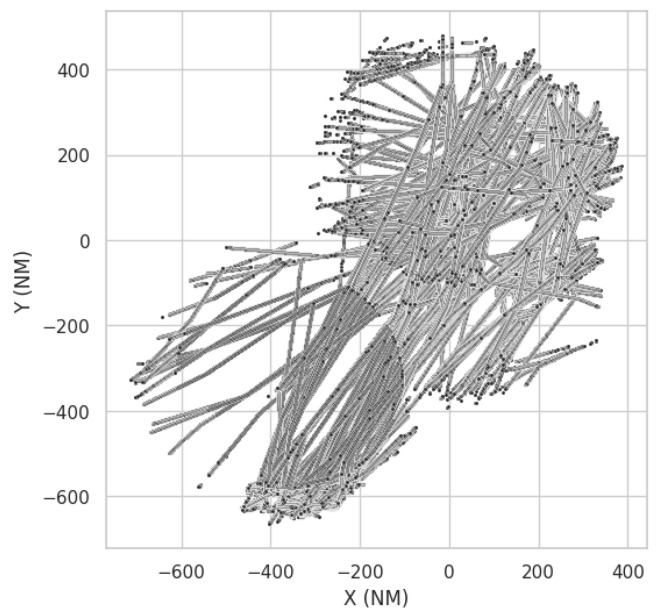
Plot of points on Surveillance Network dataset.

This dataset is formed by real surveillance reports of a control area, with all the sensors available to the Air Traffic Control (ATC). It is an example of the type of data that would be used to perform a performance evaluation of the surveillance system and represents the target environment on which the proposed model would be deployed. Figure 5 showcases this dataset.

#### 3.4.3. Dataset 3: TMA with In-Flight and Ground Sensors

**Figure 6:**
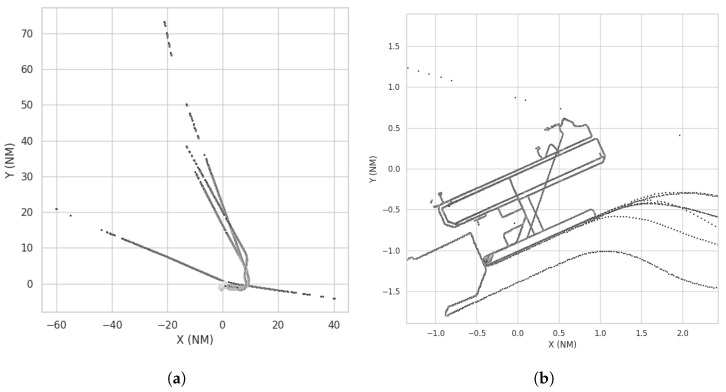
Detailed view of the airport area. (**a**) Flights in TMA Dataset. (**b**) Detailed view of the airport area.

This dataset was recorded on a TMA of a Spanish airport. It has a limited number of flights taking off, landing, and crossing the TMA with reports from different sensors, namely ADS-B, SSR RADAR, and SMR. This dataset has fewer flights, but it is important for the performance evaluation of the proposed model as it contains ground and air reports from both cooperative and non-cooperative sensors, serving as a benchmark for airport operations. Figure 6 shows a global and detailed view of this dataset.

### 3.5. M-SIOTA Training

As stated in Section 3.3, the core of the M-SIOTA algorithm is a DNN model that provides a ConfidenceScore for a given pair of track and report, estimating the suitability of the match.

**Figure 7:**
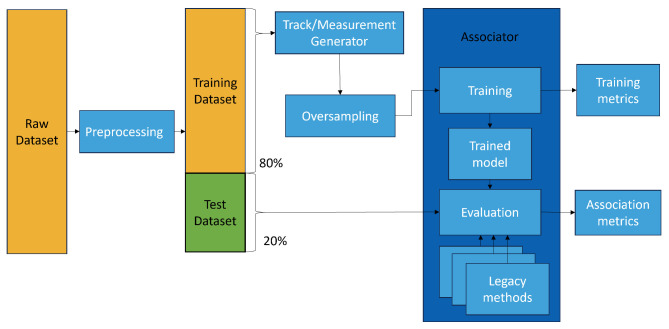
Model training and evaluation process.

The training and evaluation process starts by preprocessing the raw data and splitting them 2-fold, into a training and validation set and an evaluation set. Then, the training and validation sets are treated and augmented before training the algorithm. From the training of the neural networks, validation metrics are obtained (see Section 3.5.3). Then, with the trained model, an evaluation is made by comparing the association performance with legacy algorithms. Figure 7 shows this process.

As described in Section 3.4, the DNN model is trained with data from several sources: (1) from readily available ADS-B receptions crowd-sourced by OpenSky, (2) from a surveillance network, and (3) from an airport TMA.

Each dataset has different properties that separate it from the rest, such as the number of sensors available, their underlying technologies, and detection frequency. Because of this, the model was trained and evaluated separately for each dataset.

The OpenSky and Surveillance Network datasets were split into training and test sets by target address, with 80% of the flights used for training and the remaining left for the evaluation stage.

For the TMA dataset, as fewer targets were available, the dataset was split randomly point by point with the same 80%/20% ratio.

In the following sections, this paper goes into detail about the dataset generation and training process and the results obtained by the model.

Through this training, the DNN model learns to estimate a ConfidenceScore from the known identities on the datasets.

#### 3.5.1. Preprocessing

As each of the sensors used in this paper has a different frame of reference for their position reports, the first step is to transform the positions from the local frame of reference of each sensor to a common reference plane.

This common reference plane is defined as a Cartesian plane centred on the average longitude and latitude from the detections that are expressed on a geodetic reference system. This is used as a shorthand approach, given that the coverage of the different sensors is expected to be over the same general area.

ADS-B andWAM sensors report the positions inWorld Geodetic System 1984 (WGS84) coordinates and are transformed into the common reference plane. However, RADAR sensors report the positions in range and azimuth, which are converted to a local Cartesian plane centred on the position of the sensor and tangent to that position. Then, knowing the position of the sensor in geodetic coordinates, the detections are projected onto the geodetic reference plane and, lastly, onto the common reference plane.

#### 3.5.2. Training Data Generation

The training data are generated by creating samples of compatible and non-compatible association candidates, so the DNN model is able to learn to differentiate between correct and incorrect associations. The feedback for the learning process is obtained by comparing the estimated CompatibilityScore to the correctness of the association.

The identity of the target is obtained from the target address as received by the cooperative surveillance sensors. The dataset contains both identified and non-identified data points. Reports that do not include identification are excluded from training, as the ground truth cannot be established without identification. After excluding non-identified reports, tracks are formed by associating the reports with the same target identity. After the base track is formed, the tracks are split if there is a long gap in time between reports. Long gaps are sections of a track without a valid target report that last n sensor update intervals, in our case n=3 [46]. This long gap is defined by

(9) $$t_{thr}=\min\left\{\max\left\{3T_{i}\right\},30\;s\right\}$$

with $T_{i}$ as the period of each of the sensors under analysis.

The actual period was not available for all sensors under all datasets, so the following assumptions were made based on the expected worst-case period of each sensor [36,47]:

(10) $$\begin{array}{c} T_{ADSB}=1\;s\\ T_{MLAT}=1\;s\\ T_{RADAR}=15\;s \end{array}$$

Then, the observations are constructed for each of the points in each track. This observation contains the previous *n* reports on the track, with n being the number of inputs for the Neural Network (NN) or look-back, in our case, 10. If the track is shorter than *n*, the observation is filled with NaN values for the missing points. Each track observation is then compared to all posterior reports for compatibility, that is, all reports received after the $t_{track[i]}$ and that comply with the following limits:

(11) $$t_{thr}<t_{R}-t_{track[i]}$$

(12) $$dist_{thr}<\sqrt{{\left(x_{track[i]}-x_{R}\right)}^{2}+{\left(y_{track[i]}-y_{R}\right)}^{2}}$$

with *x* and *y* being the position in the Cartesian plane for the *i*-th report in the track and the current report to be compared, respectively, and $t_{thr}$ defined by Equation (9) and $dist_{thr}=10$ km.

These thresholds are used to select the reports that are close enough to the track to be considered for the association. This limits the number of data points for training to the most relevant ones.

If the observation complies with the limits described above, it is included in the training dataset. From each observation in the dataset, the following information is gathered:

- Position in *x*, *y*, and *z*.
- Time of applicability (*t*).
- Target address.

Then, the labels are generated by comparing the identities of the report and the track to which it is being compared. This creates an array of *Compatibility Scores* that the neural network will try to estimate.

**Figure 8:**
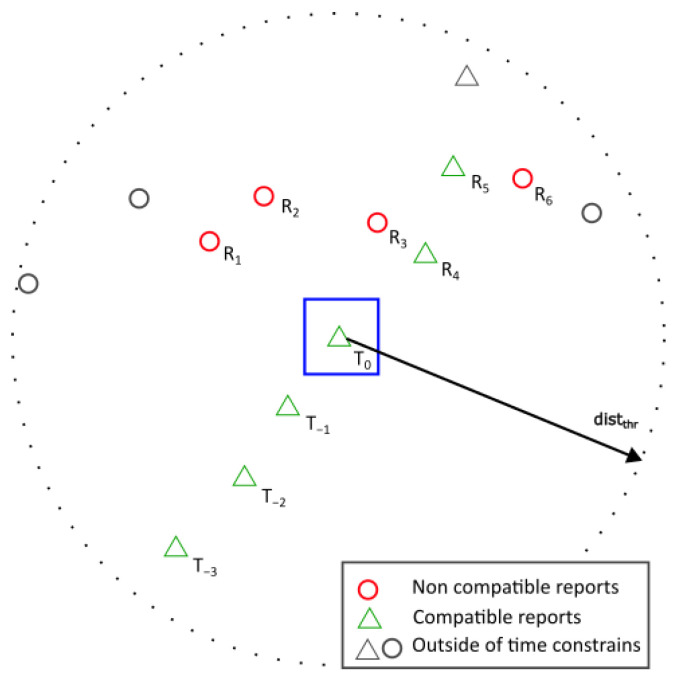
Training data generation example for a look-back of 4.

Figure 8 and Table 5 show an example of the generation of the training dataset from point $T_{0}$ of a track. Green triangles represent reports that belong to the current track, while red circles represent reports from other tracks within the time and distance constraints for generation. The observations shown in Table 5 are created by subtracting the candidate report ($R_{i}$) from each of the points on the track (*T*) in *x*, *y*, *z*, and *t*, and the corresponding label is assigned by checking if the target address from $T_{0}$ matches $R_{i}$. This process is repeated for all reports in all tracks and merged together to create the training and validation dataset.

In Figure 8, two crossing trajectories are shown. Reports R4 and R5 belong to the current track *T* and are assigned a CompatibilityScore of 1, while reports $R_{1}$, $R_{2}$, $R_{3}$, and $R_{6}$, belonging to a different target, are assigned a CompatibilityScore of 0.

Now that the dataset is generated, it needs to be normalised and balanced for proper neural network training.

**Figure 9:**
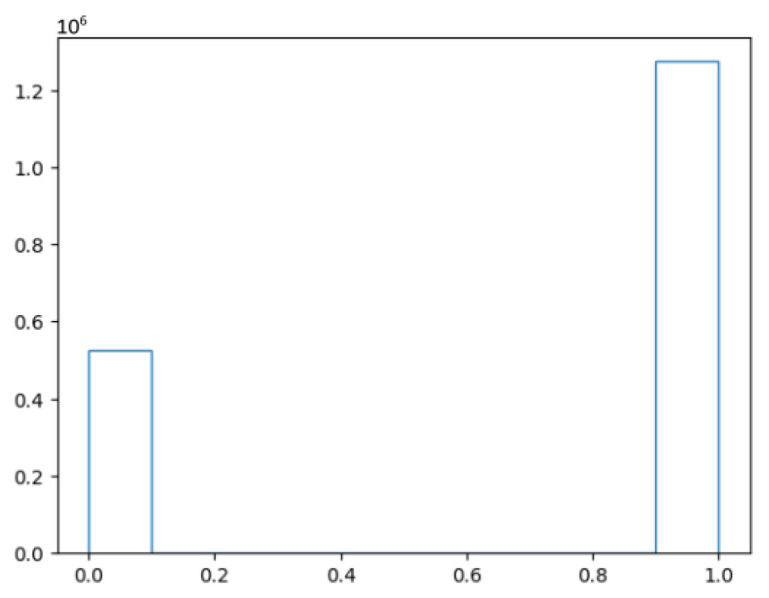
Number of compatible (1) and not compatible (0) points in the dataset.

First, the issue of balance is addressed. As the dataset is created by looking at the reports that come after a point in the track and within a certain distance, most of the reports are expected to belong to the same target. Figure 9 shows the distribution of classes between compatible (1) and incompatible (0) when applying this approach to the Surveillance Network dataset.

The dataset can be balanced before training to avoid skewed learning towards the majority class [48]. In order to solve this issue, random oversampling is used to duplicate points from the under-represented class, which, in this case, is non-compatible.

Random oversampling helps the training process; however, simply duplicating the data from the under-represented class may result in over-training of the model towards learning the duplicated points and reduce its generalisation capabilities. This can be mitigated by applying the Random OverSampling Examples (ROSE) technique as described in [49]. This technique applies a noise profile to the resampled data points to mitigate the effects of over-training on duplicated points.

Then, the dataset is standardised to be within the [−1, 1] range. In this process, each of the features is scaled by applying the following formula:

(13) $$X_{scaled}^{\prime }=\frac{X}{\mu_{X}}-\sigma_{X}$$

with $\mu_{X}$ and $\sigma_{X}$ being the average and standard deviation of feature *X*, respectively.

It was then limited to the [−1, 1] range. This standardisation was performed using the scikit-learn libraries version 1.4.1 [50].

The values of $\mu_{X}$ and $\sigma_{X}$ were stored for each feature for later use in the test dataset.

#### 3.5.3. DNN Model Performance Evaluation

The classification performance of the model was evaluated through a k-fold cross-validation procedure on the training dataset, with k=5. This process consists of splitting the training dataset into *k* independent folds. Then, the model is trained using k−1 folds and evaluated on the held back fold. The process is repeated k times, each with a different fold held back for evaluation.

Cross-validation ensures that the performance obtained is not biased due to the train/validation split and allows the evaluation of the model with data it has not seen, proving its generalization capabilities.

As the ratio of compatible and non-compatible reports in the dataset is important for our model, a stratified fold was used. The stratification ensures that the same ratio of compatible and non-compatible cases is present in each fold [51].

The metric used to evaluate the performance of the model is the *F*1 score. This score is a commonly used metric for classification that balances the effect of precision and recall metrics. This relationship is defined in the following equations:

(14) $$F1\mathrm{Score}=\frac{2\times \mathrm{Precision}\times \mathrm{Recall}}{\mathrm{Precision}+\mathrm{Recall}}$$

(15) $$Recall=\frac{TruePositives}{TruePositives+FalseNegatives}$$

(16) $$Precision=\frac{TruePositives}{TruePositives+FalsePositives}$$

Table 6 contains the results of the cross-validation applied to each dataset.

The results obtained show that the performance of the model is adequate, with F1 scores greater than 0.6 for all datasets. The effect of the fold selection in the datasets is greater on the TMA dataset, as it has fewer data points and a more heterogeneous behaviour (ground and air targets). The evaluation of the association performance of the algorithm as a whole is presented in the next section.

## 4. Experiments and Results

To demonstrate the end-to-end association performance of M-SIOTA, we performed association experiments with the different datasets and compared the results with the JPDA and GNN association algorithms [8,9]. The algorithm was also compared with the Opportunity traffic association tool SASS-C where applicable.

As stated in Section 3.3, the opportunity traffic datasets are split by 80%/20% for neural network training and testing, respectively, using the latter for this benchmark.

### 4.1. Metrics

Various metrics for the evaluation of association algorithms have been proposed in the literature [52,53,54,55,56,57]. For the evaluation of our algorithm, we have selected metrics that reflect the association performance, prioritizing it over localisation or detection metrics, as the objective of the algorithm is to provide quality measurement–target associations from a set of predefined detections. The CLEAR MOT metrics [52] present the multiple object tracking accuracy (MOTA) metric for accurate association performance measurements [58]. MOTA metrics are used in benchmarks such as the Multiple Object Tracking (MOT) Challenge [59]. In the evaluation, we display the MOTA metric, the number of misses and mismatches, and their rates.

These metrics require a ground truth for the evaluation. To create this ground truth in the opportunity traffic datasets, the target identity of 40% of the identified measurements in the datasets is hidden during association and is later used to validate the associations produced by the different methods. This ratio of identified vs. non-identified measurements is similar to the usual air surveillance system.

### 4.2. Evaluation Results

The results of this evaluation applied to the different datasets are displayed in Table 7.

**Figure 10:**
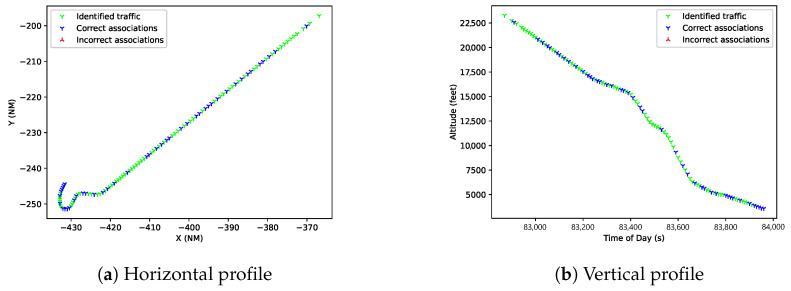
Horizontal and vertical profile of track with identified reports in green and non-identified reports in blue.

First, in the OpenSky dataset, the performance of the proposed algorithm is very similar to the GNN and JPDA. This is expected as the scenario is quite homogeneous: commercial aviation in flight phase using only ADS-B sensors. Although there is no clear improvement, this validates the application of M-SIOTA on association problems with a single sensor and no kinematic model for the target available. Figure 10 shows an example of the resulting trajectories after association with the M-SIOTA algorithm.

It is also important to remark that there is a trade-off between miss and mismatch rates between the algorithms. As the use for the associated trajectories will be to calculate error profiles of sensors and estimate their performance, it is preferable to miss more detections than to associate incorrectly in the early stages [60] and thus a lower number of mismatches is desirable.

In more complex scenarios such as the Surveillance Network, the M-SIOTA algorithm improves performance over the baselines. This improvement can result in more data being available for the performance evaluation, especially for PSR. When used on this dataset, the Opportunity Traffic Reconstructor (OTR) of SASS-C is not able to associate 11.6% of the measurements, mainly from PSR sensors. These misses are usually completed with further association processes after sensor biases are estimated and the reports are corrected for such biases. This bias correction uses the associated tracks to estimate the error profile of each sensor. An improvement in the initial association ratio can produce a more faithful bias estimation and enhance further stages of the PM process.

The TMA area is an especially complicated case for association algorithms, as it contains ground and in-flight data. This case is of special interest, as the automated merging of ground and flight data allows for the evaluation of the performance of airport surveillance systems. The GNN cannot work effectively with this kind of scenario, as shown in the results. Abrupt changes in MoF and the high accelerations and decelerations that occur during take-off and landing, combined with the close proximity of the targets on the ground, make the association of trajectories extremely difficult for traditional algorithms. The JPDA improves on the performance of the GNN, but it still has a relatively high ratio of mismatches.

The M-SIOTA algorithm displays a significant improvement over the previous algorithms in this scenario and is able to perform an adequate association of both ground and flight sections, allowing for gate-to-gate analysis of the trajectories and surveillance systems.

**Figure 11:**
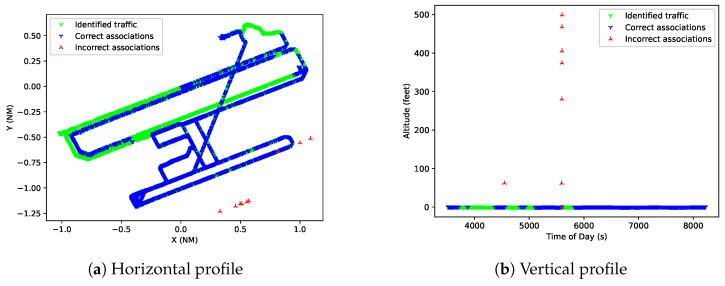
Horizontal and vertical profile of ground track with identified reports in green and non-identified reports in blue.

Figure 11 shows the association of a ground track along the airport, where the runways, taxiways, and service roads are clearly recognisable.

**Figure 12:**
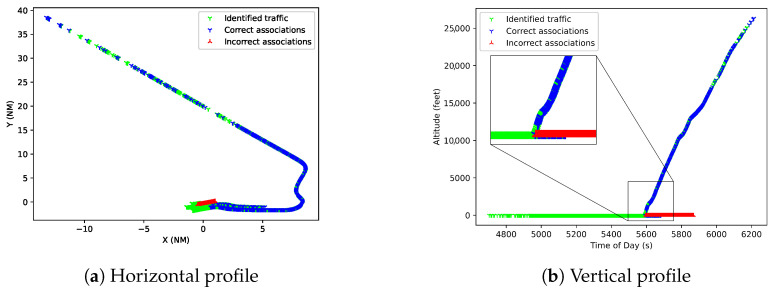
Horizontal (**a**) and vertical (**b**) profile of take-off track with identified reports in green, non-identified reports in blue, and incorrectly associated reports in red.

In Figure 12, two factors are important. First, during the taxi phase, several points are incorrectly associated with the aircraft. It is important to remark on the difficulty of association in this phase as the targets are extremely close, and current association algorithms struggle to process these reports. Second, after take-off, the vertical profile splits with correct associations (in blue) increasing in elevation and on 0 elevation. This is because some sensors, such as surface radar, do not provide altitude information and are represented in the data as a 0 elevation value. These reports belong to the same target and show that the M-SIOTA algorithm can handle sensors with and without altitude information and correctly associate the reports for both while still using altitude information when available to improve estimations. It is also a source of association errors, as several points on the ground are also associated to this track (in red).

There is still room for improvement on this front, mainly due to the relatively small size of this dataset, which limits the capabilities of the learning model. With more data of similar characteristics, an improvement in the model’s performance is expected.

### 4.3. Ablation Study

In order to understand the contribution of each of the parameters and modules of the M-SIOTA algorithm, an ablation study has been performed. The study consists of a series of experiments systematically varying several parameters and disabling some of the model’s modules. Then, the algorithm is trained and evaluated. The ablation study was performed in accordance with published research in similar applications [61,62]. The variations of the M-SIOTA algorithm are trained with the same splits as in the previous experiment and compared against the proposed version. The Surveillance Network dataset was used for this analysis.

During the tuning process of the algorithm, several parameters were detected that had a high impact on the algorithm, namely, (1) the number of dense/dropout blocks used to build the DNN model, (2) the usage of dropout layers, (3) the usage of the ROSE oversampling technique for training, and (4) the minimum association threshold.

Although the MOTA metric remains similar for most of the parameters, the experiments show an increase in the number of mismatches as the size of the model is reduced from 10 to 8 and 6 dense/dropout blocks. The performance of the model also worsens when the ROSE is removed, as the imbalance of compatible and non-compatible data points hinders the training process.

The inclusion of dropout layers to reduce the impact of over-training also appears to be effective. Even with the early stopping of 5 epochs, the model seems to overfit if the dropout layers are removed.

Lastly, the minimum association threshold is the minimum ConfidenceScore required to associate a report with a track as used in Algorithm 1. As the results in Table 8 show, lowering this threshold leads to lower misses as the number of associations is higher, but the quality of the associations is reduced as the number of mismatches increases. The inverse occurs when the threshold is increased, trading-off misses for mismatches. Depending on the current requirements, this threshold may be tuned to either ensure only correct associations are made or associate as much as possible (with lower confidence). For surveillance performance evaluations, fewer mismatches are preferable when association performance is similar [60].

## 5. Conclusions

This paper has proposed an architecture and working model for the association of opportunity traffic with the M-SIOTA algorithm. M-SIOTA is able to perform the correlation of real-world data that include TMA and en-route trajectories, both ground and air data, and can handle multiple concurrent sensors based on different technologies (i.e., RADAR, WAM, and ADS-B), while not requiring previous knowledge of the flight characteristics of the targets or error profiles of the sensors. This will enable the creation of a new generation of Surveillance Performance Monitoring tools that can adapt to the changes in the airspace characteristics as they happen, reducing development time and improving the safety and efficiency of the airspace.

The association model has been validated with several opportunity traffic datasets from different geographical areas and with different sensor mixes, and it maintained acceptable association performance regardless of the underlying conditions. In the validation process, the tool was compared with the industry standard tool SASS-C in the compatible dataset, displaying a significant improvement in the ratio of missed reports. The ablation study demonstrates that specific parameters and techniques impact performance. These findings point to promising directions for future work, including exploring alternative structures for neural networks. Such targeted investigations could lead to substantial improvements in association performance.

The work in this paper serves as a proof of concept that opens the exploration of the application of state-of-the-art modelling and machine learning techniques to the rest of the stages of the performance evaluation, namely the reconstruction of trajectories and the estimation of the noise and bias characteristics of the sensors.

## Figures and Tables

**Table 1:** Sensor hierarchy.

Uniquely identified	ADS-B reports
	Mode S RADAR reports
	Mode S WAM reports
Non-identified	Mode A RADAR reports
	Mode A WAM reports
	PSR RADAR reports

**Table 2:** Layer parameters.

	Type	Neurons	Rate	Activation
Input layer	Input	40	-	-
Block	Dense	512	-	ReLU
	Dropout	-	20%	-
Output layer	Dense	1	-	Sigmoid

**Table 3:** DNN hyperparameter table.

Hyperparameters	
Batch size	256
Epochs	150
Early stopping	5
Optimizer	Adam
Learning rate	0.0001
Loss function	Binary crossentrpy

**Table 4:** Dataset statistics.

Dataset	No. of Reports	No. of Unique Identifiers	% Identified	# Sensors
OpenSky	2,318,343	11,195	100%	1
Surveillance Network	2,979,716	759	67.3%	6
TMA	147,848	9	15.6%	3

**Table 5:** Training data (X) and labels (Y) generated for the report $T_{0}$.

X				Y
$\Delta_{T_{-3},R_{1}}$	$\Delta_{T_{-2},R_{1}}$	$\Delta_{T_{-1},R_{1}}$	$\Delta_{T_{0},R_{1}}$	$cmp(T_{0},R_{1})=0$
$\Delta_{T_{-3},R_{2}}$	$\Delta_{T_{-2},R_{2}}$	$\Delta_{T_{-1},R_{2}}$	$\Delta_{T_{0},R_{2}}$	$cmp(T_{0},R_{2})=0$
$\Delta_{T_{-3},R_{3}}$	$\Delta_{T_{-2},R_{3}}$	$\Delta_{T_{-1},R_{3}}$	$\Delta_{T_{0},R_{3}}$	$cmp(T_{0},R_{3})=0$
$\Delta_{T_{-3},R_{4}}$	$\Delta_{T_{-2},R_{4}}$	$\Delta_{T_{-1},R_{4}}$	$\Delta_{T_{0},R_{4}}$	$cmp(T_{0},R_{4})=1$
$\Delta_{T_{-3},R_{5}}$	$\Delta_{T_{-2},R_{5}}$	$\Delta_{T_{-1},R_{5}}$	$\Delta_{T_{0},R_{5}}$	$cmp(T_{0},R_{5})=1$
$\Delta_{T_{-3},R_{6}}$	$\Delta_{T_{-2},R_{6}}$	$\Delta_{T_{-1},R_{6}}$	$\Delta_{T_{0},R_{6}}$	$cmp(T_{0},R_{6})=0$

**Table 6:** Cross-validation results for DNN model.

Dataset	Validation Loss		F1 Score	
	avg	σ	avg	σ
OpenSky	0.763	0.026	0.737	0.118
Surveillance Network	0.634	0.367	0.685	0.080
TMA	0.902	0.89	0.743	0.179

**Table 7:** Evaluation results.

Dataset	Algorithm	Misses ↓	Mismatches ↓	MOTA ↑	Miss Ratio ↓	Mismatch Ratio ↓
OpenSky	M-SIOTA	323	**75**	95.93%	3.43%	**0.65%**
	JPDA	213	86	**97.42%**	**1.83%**	0.74%
	GNN	**272**	121	96.61%	2.34%	1.04%
Surv. net.	M-SIOTA	**875**	**35**	**99.46%**	**0.51%**	**0.02%**
	JPDA	8620	1490	94.05%	5.07%	0.88%
	GNN	12240	1610	91.85%	7.20%	0.95%
TMA	M-SIOTA	**397**	314	**76.13%**	**13.33%**	10.54%
	JPDA	398	546	68.31%	13.36%	18.33%
	GNN	1198	**0**	59.79%	40.21%	**0.00%**

↑ The upwards arrow indicates increasing performance with increasing values. ↓ The downwards arrow indicates increasing performance with decreasing values. **Bold** values highlight the best result for the metric in each dataset. The same notation is used in the following tables.

**Table 8:** Ablation study results for the M-SIOTA algorithm.

Variation		Misses ↓	Mismatches ↓	MOTA ↑	Miss Rate ↓	Mismatch Rate ↓
Base		875	35	99.46%	0.02%	0.51%
No. of blocks	8	667	39	99.58%	0.02%	0.39%
	6	685	110	99.53%	0.06%	0.40%
Dropout	No	441	1642	98.77%	0.97%	0.26%
Rose	No	507	169	99.60%	0.10%	0.30%
Assoc. thr.	0.4	670	125	99.53%	0.07%	0.39%
	0.6	11,220	0	93.40%	0.00%	6.60%

## Data Availability

The original contributions presented in the study are included in the article; further inquiries can be directed to the corresponding author. The implementation of the algorithm described in this paper is available at https://github.com/JoaquinVico/M-SIOTA (accessed on 29 January 2025).
